# The bright side and dark side of trust: The mediating effect of franchisor trust on performance

**DOI:** 10.1002/mde.3097

**Published:** 2019-10-27

**Authors:** Dana Minarikova, Nada Mumdziev, Michele Griessmair, Josef Windsperger

**Affiliations:** ^1^ Faculty of Business, Economics, and Statistics Vienna Austria; ^2^ Visiting Research Fellow, Sir Zelman Cowen Centre Victoria University Melbourne Australia; ^3^ Business and Management Department Webster Vienna Private University Vienna Austria; ^4^ Supported by an Erwin Schrödinger Fellowship from the Austrian Science Fund (J 3866‐G27)

## Abstract

Previous literature has not examined the dual role of trust in franchise relationships. We extend the franchise and relational governance literature by showing that trust has both a “bright side” and a “dark side” in franchisor–franchisee relationships. Based on transaction cost and knowledge‐based reasoning, we argue that intangible knowledge assets and environmental uncertainty have an indirect effect on performance via trust, due to its relational risk and knowledge exchange effect. Using data from the franchise sector in Germany, we show that trust positively mediates the impact of intangible knowledge assets and negatively mediates the impact of environmental uncertainty on franchisor performance. The first effect refers to the “bright side” of trust showing that intangible brand name assets increase trust which, in turn, has a positive effect on performance. Conversely, the second effect refers to the “dark side” of trust highlighting that environmental uncertainty diminishes trust resulting in a negative effect on performance.

## 1 INTRODUCTION

This study reveals the dual role of trust in franchisor–franchisee relationships. Trust can improve performance, but it can also hurt performance in network relationships (Villena, Revilla, & Choi, 2011). Although trust has been frequently examined in previous franchise literature (e.g., Bordonaba‐Juste & Polo‐Redondo, 2004; Calderon‐Monge & Pastor‐Sanz, 2017; Chiou & Droge, 2015; Cochet, Dormann, & Ehrmann, 2008; Croonen, 2010; Croonen & Brand, 2013; Dahlstrom & Nygaard, 1995; Davies, Lassar, Manolis, Prince, & Winsor, 2011; Dickey, Harrison McKnight, & George, 2008; Eser, 2012; Gorovaia & Windsperger, 2011; Grace, Frazer, Weaven, & Dant, 2016; Griessmair, Hussain, & Windsperger, 2014), to the best of our knowledge, this research has not analyzed both the performance‐enhancing and performance‐reducing effect of trust in franchising networks.

Several authors have examined the role of trust in franchising context by applying the franchisee or franchisor perspective. Studies that investigated the determinants and consequences of trust from the franchisee perspective mainly focus on the effects that franchisors' trustworthiness has on franchisees' compliance, cooperativeness, and termination rate (Bordonaba‐Juste & Polo‐Redondo, 2004; Chiou & Droge, 2015; Cochet et al., 2008; Croonen & Brand, 2013; Dahlstrom & Nygaard, 1995; Davies et al., 2011; Dickey et al., 2008). On the other hand, studies that investigated trust from the franchisor perspective analyze how trust affects governance of franchise networks, such as allocation of decision rights and the knowledge transfer strategy (Gorovaia & Windsperger, 2011; López‐Fernández & López‐Bayón, 2011; Mumdžiev & Windsperger, 2013). Most of the empirical studies confirm that trust reduces transactional risk, supports an open way of knowledge sharing, facilitates decentralization of decision making, and has a positive impact on performance of franchise systems (Bordonaba‐Juste & Polo‐Redondo, 2004; Dahlstrom & Nygaard, 1995; Dickey et al., 2008; Hertz, Hutzinger, Seferagic, & Windsperger, 2016). Franchisors that trust their franchisees can rely on their efficient way of operating, preserving high‐level product and service quality as well as unified image across the chain (Chiou & Droge, 2015; Dahlstrom & Nygaard, 1995). In addition, Calderon‐Monge and Pastor‐Sanz (2017) provide some evidence that the performance effect of trust varies with the network structure (e.g., single‐unit franchising or multi‐unit franchising).

Therefore, most of the previous studies in franchising research focus on the positive effect of trust that results from mitigating relational risk and increasing transaction value by facilitating knowledge sharing between the partners. These effects constitute the “bright side” of trust in network relationships (Noordhoff, Kyriakopoulos, Moorman, Pauwels, & Delleart, 2011; Zand, 1972). However, trust may also have a “dark side” resulting from its negative effects on knowledge exchange and increasing relational risk (Anderson & Jap, 2005), especially under high environmental uncertainty due to over‐embeddedness (Uzzi, 1997). As trust strengthens the ties between franchisor and franchisees, they are less capable or motivated to consider the negative consequences of important changes of the external environment for their internal network relationship. In this case, trust may become a source of “strategic blindness” by restricting the information flow and inhibiting the exploration of novel information (McEvily, Perrone, & Zaheer, 2003, 97; Zheng Zhou, Zhang, Sheng, Xie, & Bao, 2014). Therefore, highly embedded franchisor–franchisee relationships do not adequately respond to challenges posed by an uncertain external environment (Krishnan, Martin, & Noorderhaven, 2006). Focusing on the “bright side” of trust, prior research neglected these potential negative consequences of trust in franchise relationships.

Adressing this gap, we examine the mediating role of trust in affecting the performance of franchise firms via environmental uncertainty, transaction‐specific investments, and intangible system‐specific and brand name assets. Whereas environmental uncertainty and transaction‐specific investments reflect the transaction cost perspective, the proposed relationships via intangible system‐specific and brand name assets are rooted in the knowledge‐based view of franchise relationships. Specifically, we argue that trust positively mediates the impact of intangible knowledge assets (the “bright side”) and negatively mediates environmental uncertainty (the “dark side”) on franchisor performance. The proposed mediation hypotheses are tested with data from the German franchise sector by estimating an intervening variable model employing the approach proposed by Hayes and colleagues (Hayes, 2009, 2013; Preacher & Hayes, 2004). The results support the hypothesized mediating role of trust, and we find significant indirect effects of environmental uncertainty and intangible brand name assets on franchisor performance via trust.

This study contributes to the franchise literature by showing that trust has a dual role, that is, a “bright side” and a “dark side” in franchising networks. As facilitator of knowledge transfer, it has a positive effect on franchisor performance under increasing intangible knowledge assets, and as relational governance mechanism, it has a negative effect on performance under high environmental uncertainty. These empirical results have important managerial implications for the franchisors. Our findings indicate that trust is a central relational factor positively affecting the performance of the franchise system, particularly when intangible brand name assets are involved. On the other hand, trust has a detrimental effect on performance, particularly under high environmental uncertainty. Thus, when confronted with high environmental uncertainty and high intangible brand name assets, it is paramount for franchisors to take into account the opposite performance effects of the “bright side” and “dark side”of trust.

The paper is organized as follows. In Section 2, we elaborate our theoretical approach and develop the hypotheses. Section 3 provides the empirical results, followed by the discussion and conclusion in Section 4.

## 2 RESEARCH FRAMEWORK AND HYPOTHESES

**Figure 1:**
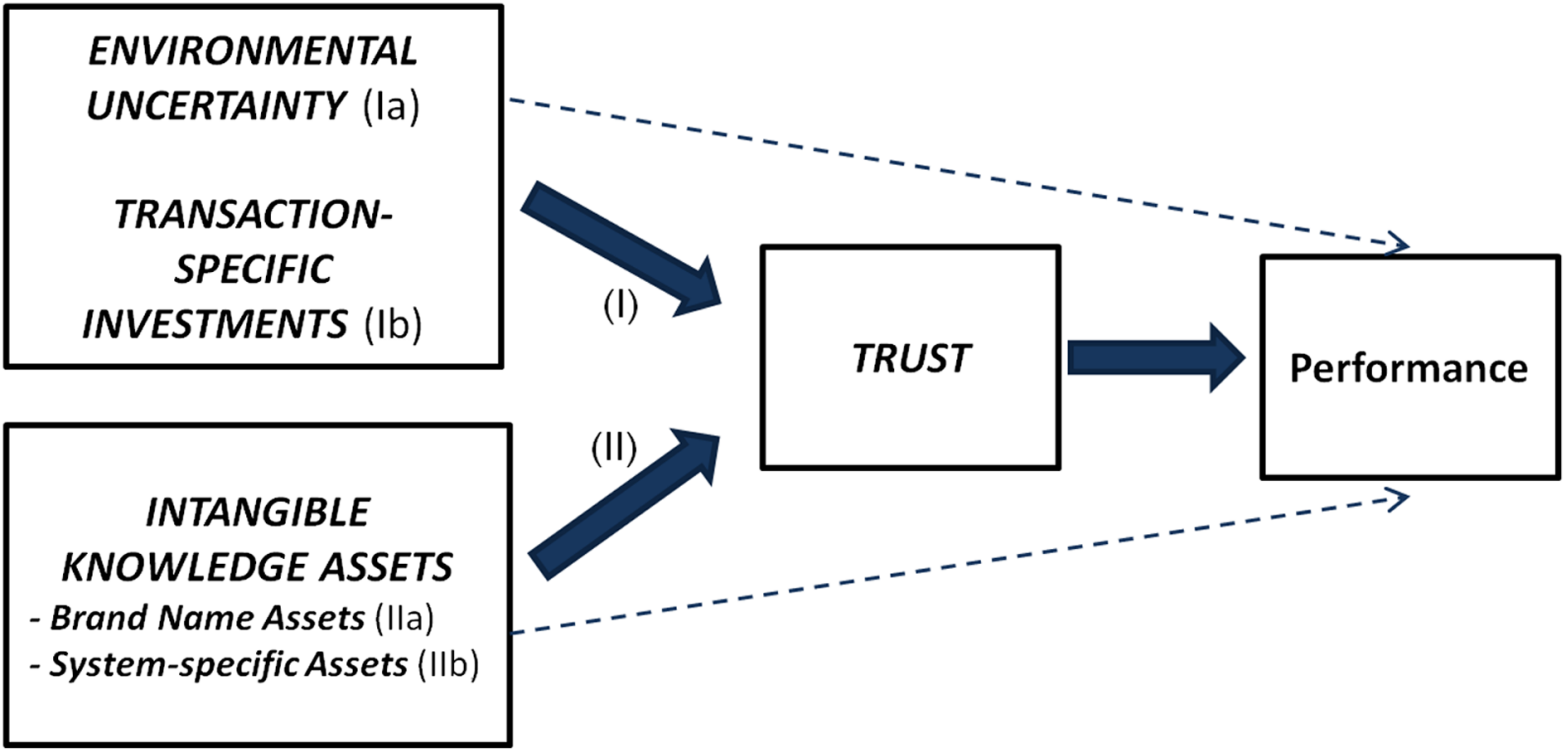
Conceptual Framework [Colour figure can be viewed at http://wileyonlinelibrary.com]

The central focus of our research model is to test the mediating role of trust by hypothesizing that the transaction cost variables (environmental uncertainty and transaction‐specific investments) and the knowledge‐based variables (intangible system knowhow and brand name assets) exert not only a direct effect on performance but also affect performance indirectly via trust (see Figure 1).

In a first step, we provide an explanation of the relationship between trust and performance. Second, we establish the antecedents of trust based on transaction cost and knowledge‐based reasoning and, as a consequence, the indirect effects the antecedents have on performance. We distinguish two indirect effects via trust on franchisor performance (see Figure 1): (I) The relational risk effect is associated with environmental uncertainty (Ia) and transaction‐specific investments (Ib), and (II) the knowledge exchange effect is associated with the intangibility of knowledge assets, such as brand name assets (IIa) and system‐specific knowhow (IIb).

**Figure 2:**
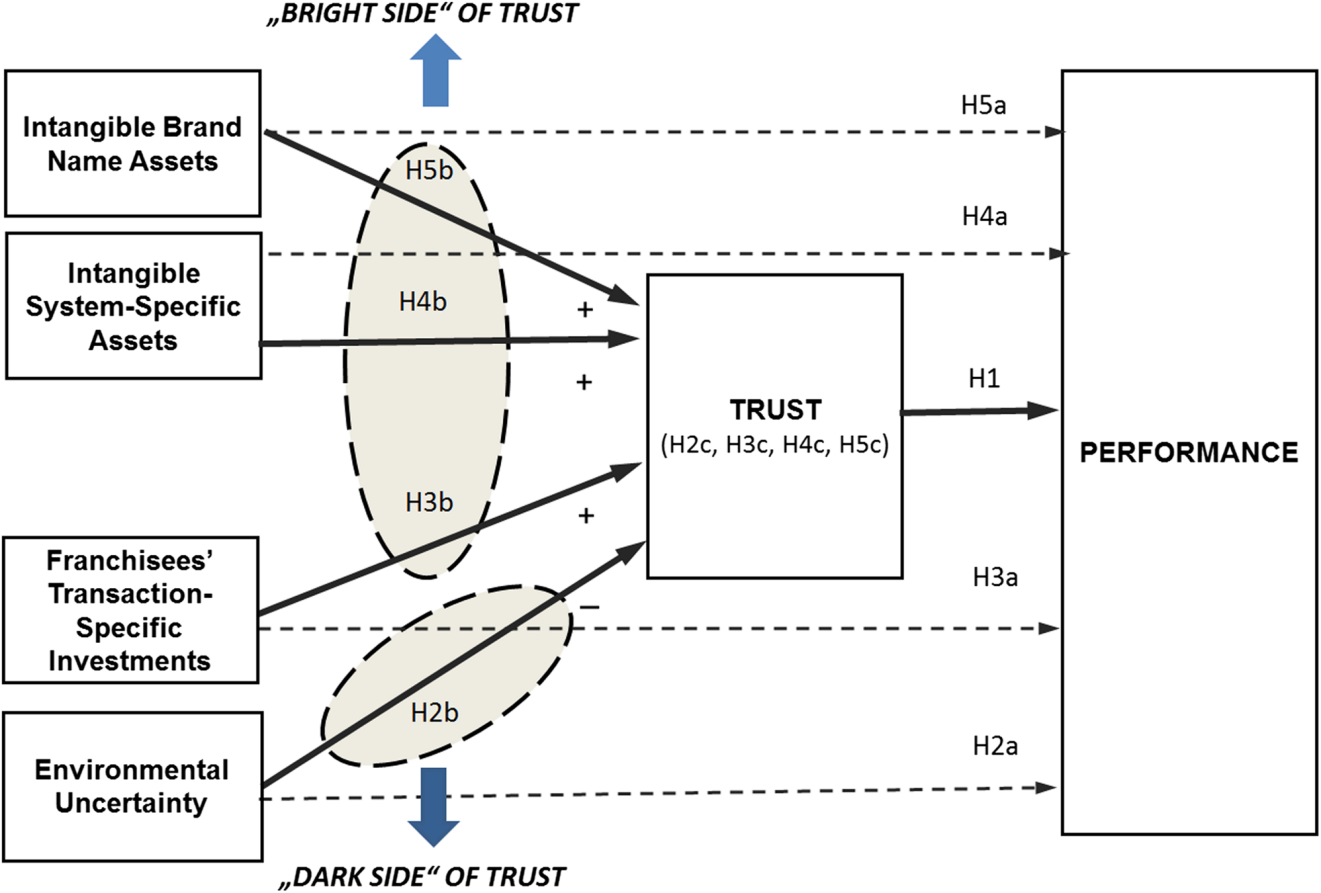
Research Model [Colour figure can be viewed at http://wileyonlinelibrary.com]

The research model of the mediating effect of trust on franchisor performance is summarized in Figure 2. The “bright side” of trust refers to the positive impact of the intangible knowledge assets and transaction‐specific investments via trust on performance, and the “dark side” of trust refers to the negative impact of environmental uncertainty via trust on performance.

### 2.1 Direct effect of trust on performance

Due to the multidisciplinary focus of trust research, the trust concept is very heterogeneous (Williamson, 1993; Palmatier, Dant, & Grewal, 2007; Poppo, Zhou, & Li, 2016; Yamagishi & Yamagishi, 1994). In line with Mayer, Davis, and Schoorman (1995), franchisor trust can be defined as the franchisor's willingness to be vulnerable to the actions of the franchisee based on the expectation that the franchisee will perform a particular action important to the franchisor, irrespective of the ability to monitor the network partner. Therefore, trust represents the franchisor's perception of the franchisee's trustworthiness. In this study, we apply the concept of knowledge‐based trust developed by Yamagishi and Yamagishi (1994). According to Yamagishi and Yamagishi (1994), trust can be differentiated, among others, in general and knowledge‐based trust. This concept of trust is different from general trust, which exists without having an interaction history with the trustee and is mainly influenced by the trustor's personality traits. Knowledge‐based trust refers to the perception of a partner's trustworthiness based on information accumulated over a period of time, that is, an experience‐based expectation that individuals will not behave opportunistically when having the opportunity to do so (Shapiro, Sheppard, & Cheraskin, 1992). As franchising is relational by nature, the present paper focuses on a trust concept that is based on the interaction history between franchisor and franchisee.

A number of studies show that trust has a positive impact on performance in interorganizational relationships (e.g., Barney & Hansen, 1994; Dant, 2008; Dyer, Singh, & Kale, 2008; Poppo et al., 2016; Poppo, Zhou, & Zenger, 2008). Based on the transaction cost and knowledge‐based view, we can differentiate two effects of trust on franchisor performance. Trust increases franchisor performance by reducing franchisor's relational risk, leading to lower transaction costs. In addition, trust operates as facilitator for and basis of open communication, thereby increasing knowledge sharing between the franchisor and the franchisees. A higher level of information exchange increases transaction value as relational rents (Dyer & Chu, 2000; Zajac & Olsen, 1993). Thus, in line with prior research, we hypothesize that trust has an overall positive direct effect on firm performance (see Figure 2):

Hypothesis 1: Franchisor trust has a positive effect on franchise firm performance.

### 2.2 Transaction cost view and relational risk effects of trust

From the perspective of transaction cost theory, environmental uncertainty and transaction‐specific investments influence the degree of trust franchisors have in their franchisees, which, in turn, affects the performance of the franchise system (see Figure 2).

#### 2.2.1 Environmental uncertainty

1. Direct effect on performance

According to the transaction cost theory, environmental uncertainty increases franchisor's difficulty in anticipating changes in the local market environment (Sheng, Brown, Nicholson, & Poppo, 2006). Under environmental uncertainty, organizational routines are less established, and the criteria for evaluation of results and alternatives are not very clear (Homburg, Krohmer, & Workman, 1999). Parkhe (1993) argues that perception of opportunistic behavior in the context of interfirm alliances can be amplified by the increased environmental uncertainty, which would in turn mobilize greater coordination efforts and monitoring. Higher environmental uncertainty increases the information‐processing requirements and hence the information and communication costs in the franchise network. This increase in transaction costs may lead to lower performance. In this case, the franchisor has to set up a more complex coordination and control system with a higher information processing capacity (Haleblian & Finkelstein, 1993). For example, Safón and Escribá‐Esteve (2011) show that uncertain external conditions force franchise managers to allocate more resources to reducing environmental uncertainty. We thus expect that environmental uncertainty negatively influences the performance of the franchise system.

Hypothesis 2a Environmental uncertainty has a negative effect on performance of franchise firms.

- Environmental uncertainty and the “dark side” of trust

Franchisor's perception of the environmental uncertainty is likely to influence the level of franchisor trust towards franchisees. If the franchisor is not able to assess franchisees' outcomes due to environmental uncertainty, it may become increasingly difficult to evaluate the competencies, behavior, and performance of the franchisees. Such circumstances may negatively affect the level of franchisors' perception of franchisees' trustworthiness. In addition, strong ties between franchisor and franchisees may result in „strategic blindness“(McEvily et al., 2003, 97) under high environmental uncertainty. For instance, if environmental changes would require major changes in the governance of franchising network, over‐embeddedness prevents the franchisor from adequately responding to demands of the external environment. Consequently, because franchisors' vulnerability rises when the franchisees operate in an uncertain local market environment, their level of trust is expected to be lower. We thus expect that environmental uncertainty negatively influences franchisors' trust in their franchisees. As a result, environmental uncertainty has not only a direct effect on performance (H2a) but also an indirect effect via trust. That is, the higher the environmental uncertainty, the lower the level of trust (H2b). As trust has a positive effect on performance, its diminished levels result in decreased performance. Thus, the negative effect of environmental uncertainty on performance is enhanced by its negative effect on trust (H2c).

Hypothesis 2b Environmental uncertainty has a negative effect on franchisor trust.

Hypothesis 2c Environmental uncertainty has a negative indirect effect on performance via trust.

#### 2.2.2 Franchisees' transaction‐specific investments

1. Direct effect on performance

Transaction costs theory suggests that transaction‐specific investments influence the partners' quasi‐rents (Williamson, 1985). Franchisees are required to make particular transaction‐specific investments when they enter a franchise system and open a local outlet. These investments include obtaining and adapting premises, tools and equipment, specific software or computer systems, the local advertising of launching the new business, etc. Much of the equipment and fittings is trademarked, which results in high sunk costs due to the investments in such highly transaction‐specific assets (Dnes, 1993). Transaction cost theory (Williamson, 1979, 1985) predicts that higher transaction‐specific investments are likely to increase franchisor performance due to their positive effect on the quasi‐rent stream of the network partners. As the transaction‐specific investments of franchisees increase, their quasi‐rents are likely to exceed the potential hold‐up gains from opportunistic behavior (Katsikeas, Skarmeas, & Bello, 2009; Klein, 1996). This bonding effect may motivate franchisees to behave cooperatively in order to realize high relationship‐specific quasi‐rents. Hence, the following hypothesis can be formulated:

Hypothesis 3a Franchisees' transaction‐specific investments have a positive effect on the performance of franchise firms.

- Franchisees' transaction‐specific investments and the “bright side” of trust

Higher levels of franchisees' transaction‐specific investments may also affect how franchisors perceive franchisees. The induced self‐enforcement effect can be related to lower monitoring of franchisees, relative to managers of company‐owned outlets, as well as the perception of franchisees as more trustworthy. We can therefore predict that with higher franchisees' transaction‐specific investments franchisor's perception of franchisees' trustworthiness will increase. Thus, franchisees' transaction‐specific investments have not only a direct effect on performance (H3a) but also increase trust (H3b) which in turn increases performance.

Hypothesis 3b Franchisees' transaction‐specific investments have a positive effect on franchisor trust.

Hypothesis 3c Transaction‐specific investments have a positive indirect effect on performance via trust.

### 2.3 Knowledge‐based view and knowledge exchange effects of trust

According to the knowledge‐based theory, trust increases relationship‐specific rents through improving knowledge exchange between the network partners. We propose that intangible system‐specific and brand name assets positively influence the degree of trust franchisors have in their franchisees, which, in turn, affects the performance of the franchise system (Figure 2).

#### 2.3.1 Franchisor's intangible system‐specific assets

1. Direct effect on performance

Franchisor's intangible system‐specific assets refer to the system‐specific knowhow—such as marketing knowhow, organizational knowhow, administrative knowhow, quality management knowhow, accounting knowhow, and IT‐knowhow—that is difficult to articulate and imitate, thereby driving the franchise firm's competitive advantage and performance (Erramilli, Agarwal, & Dev, 2002). A franchisor that invests in intangible system knowhow contributes to the success of the system (Bradach, 1998; Fulop, 2000) as its system knowhow represents an important input for the franchisees' operations (Monroy & Alzola, 2005). Hence, we can formulate the following hypothesis:

Hypothesis 4a Franchisor's intangible system‐specific assets have a positive effect on the performance of franchise firms.

- Intangible system assets and the “bright side” of trust

Intangible system‐specific assets refer to tacit knowledge that is embedded in the firm's employees and organizational routines and difficult to transfer to network partners (Madhok, 1997; Nonaka, 1994). Successful franchising requires that the franchisor's specific knowhow is efficiently transferred to the local network partners, whose task becomes more difficult if the knowledge assets are more intangible. Higher intangible system‐specific assets require more intensive and frequent interaction between partners. Through repeated interaction network partners learn about each other and develop personal ties (Shapiro et al., 1992), which in turn facilitates establishing trust. Hence, the more the franchisor–franchiseee relationship involves intangible system‐specific assets, the more likely a franchisor develops trust towards the franchisees, which facilitates the knowledge exchange between the two network partners. As a consequence, a higher degree of intangibility and the resulting lower degree of transferability of system‐specific knowhow is associated with a higher level of franchisor trust towards their franchisees.

In addition, we expect that trust will mediate the relation between franchisor's intangible system‐specific assets and franchise firm performance. Trust influences the transfer of knowledge when franchise contracts are incomplete due to the intangibility of knowledge assets. Therefore, if franchisor's system‐specific assets are tacit, the franchisor will develop trust as relational mechanism that enables the transfer of intangible knowledge assets to the local outlets. Thus, the positive effect of intangible system‐specific assets on performance (H4a) is enhanced by its positive effect on trust. Based on this reasoning, we formulate the following hypotheses:

Hypothesis 4b Franchisor's intangible system‐specific assets have a positive effect on franchisor trust.

Hypothesis 4c Intangible system‐specific assets have a positive indirect effect on performance via trust.

#### 2.3.2 Franchisor's intangible brand name assets

1. Direct effect on performance

Similar to the intangible system‐specific assets, brand name represents another important and essential element of intangible knowledge assets. From a knowledge‐based perspective (Nonaka, 1994; Zander & Kogut, 1995), intangible brand name assets create competitive advantage because they cannot be easily imitated by potential competitors due to their high tacitness component. Therefore, transferring intangible brand name assets to the franchise partners will lead to an increase of relationship‐specific rents, which in turn results in superior franchisor performance. Hence, we hypothesize:

Hypothesis 5a Franchisor's brand name assets have a positive effect on the performance of franchise firms.

- Intangible brand name assets and the “bright side” of trust

Franchisors' intangible brand assets signal their commitment to the relationship (Ganesan, 1994; Jap, Manolis, & Weitz, 1999; Morgan & Hunt, 1994), thereby enhancing trust between the network partners (Lui, Wong, & Liu, 2009). Thus, we hypothesize that higher intangible brand name assets, created by the franchisors, will stimulate more trustworthy behavior of franchisees, which, in turn, will increase the perception of higher franchisor trust in its franchisees. As a result, intangible brand name assets will not only have a direct effect on performance (H5a) but also positively affect performance via trust. As hypothesized above, intangible brand name assets have a positive effect on trust (H5b) which, in turn, has a positive effect on performance. Consequently, intangible brand name assets also exert an indirect effect on performance (H5c).

Hypothesis 5b Franchisor's brand name assets have a positive effect on franchisor trust.

Hypothesis 5c Intangible brand name assets have a positive indirect effect on performance via trust.

## 3 EMPIRICAL ANALYSIS

In order to test our hypotheses, we estimate an intervening variable model employing the approach proposed by Hayes and colleagues (Hayes, 2009, 2013; Preacher & Hayes, 2004). In an intervening variable model, causal variables *X*_*n*_ are postulated to exert an effect on an outcome variable *Y* through a mediating variable *M*. In the present article, we hypothesized that the knowledge‐based and transaction cost variables exert not only a direct effect on performance (path *c*) but also on trust (path *a*), which in turn affects performance (path *b*). The total effect the causal variables have on performance is composed of the direct effect (*c'*) as well as the indirect effect via trust (*ab*). As opposed to the traditional approach for modeling indirect effects (Baron & Kenny, 1986), Hayes' method allows to quantify and test the intervening effect *ab* and additionally does not suffer from power issues (Fritz & MacKinnon, 2007; MacKinnon, Lockwood, Hoffman, West, & Sheets, 2002). Our analysis is conducted in two steps (Anderson & Gerbing, 1988). First, we establish the validity of the measurement model employing confirmatory factor analysis using AMOS version 20 (Arbuckle, 2011). Second, we test the hypothesized direct and indirect relationship using PROCESS (Hayes, 2012).

### 3.1 Sample

We collected the data for the empirical analysis via self‐administered questionnaire from the German franchise sector. As a source of contacts for German franchise companies, we used a franchise guide “Franchise Wirtschaft” and the directory of the German Franchise Association. This franchise guide lists all franchise systems in Germany including data such as year of establishment, sector, and number of outlets. After removing from the list of 891 companies, those franchise systems, which had less than five outlets in Germany, were not active before 2008. This was necessary, as we wanted to make sure that the informants in the chosen companies have the relevant knowledge related to our variables. Finally, the questionnaire was sent to 491 franchise systems. The chosen respondents were selected based on their expertise and relevance to the subject under investigation (McKendall & Wagner, 1997).

The questionnaire was developed in several steps. After reviewing the literature to generate the relevant measurements and items for the variables in the model, franchise professionals (franchisors and franchise consultants) from Germany and Austria were interviewed to help refine the questions and formulations within the questionnaire and ensure the face validity of the questions. In the final step, we pretested the questionnaire on 20 Austrian franchise systems. The final sample for the study consisted only of franchise system listed in the “Franchise Wirtschaft” and the directory of the German Franchise Association. The data collected in Austrian were solely used for testing the questionnaire.

The questionnaires were mailed to the general managers of the franchise systems (franchisors) as key informants of all 491 chosen franchise systems. Our final sample was composed of 137 questionnaires (response rate was 28%). Fourteen questionnaires had to be eliminated due to missing values in the relevant variables resulting in a final sample of 123. About 65% of the companies are from the services sector, and the remaining 35% are product franchising companies. On average, 22.66% of the outlets are company‐owned. The average age of the franchise system is 11.2 years. The average initial fees are 12.73 thsd EUR, and the average royalty rate (including advertising fees) is 6.37%. The average investment required by a franchisee to start a new franchised outlet is 457.92 thsd.

We tested for common method and nonresponse bias. The Harman's single‐factor test indicates that common method bias can be excluded (Podsakoff, MacKenzie, Lee, & Podsakoff, 2003). Nonresponse bias was tested by comparing the data of early and late respondents (Armstrong & Overton, 1977), and we also used available data on companies not in the sample. Independent *t*‐test revealed no significant differences regarding variables of interest (e.g., size, fees and royalties, and age; see Table 1).

**Table 1:** Estimate of nonresponse bias*

	Means, (*SD*), and counts**			
	Population	Respondents	*t*‐value	*p*‐value
Age of franchise system (years)	10.102 (8.122) *N* = 449	11.190 (8.391) *N* = 121	−1.298	.195
System size (total outlets)	112.718 (431.444) *N* = 337	155.949 (328.376) *N* = 118	0.992	.322
Advertising fee (% of sales)	1.002 (1.497) *N* = 326	0.930 (1.342) *N* = 127	−0.478	.633
Royalties (% of sales)	4.473 (6.282) *N* = 446	5.442 (7.452) *N* = 117	1.408	.16

* The measures of advertising fee and royalties were first tested by a multivariate analysis of variance to ensure independence of these variables.

** Counts differ across different measures because of missing values.

### 3.2 Measurement

This section provides a brief overview of the variables used in the analysis. A more detailed description of the reliability and validity assessments can be found in the Measurement model section. The study is based on self‐reports. Employing self‐reports is common in social sciences, particularly when “objective” data are not available or cannot be obtained. Furthermore, self‐report measures, including subjective performance measures, have already been successfully employed in management and organizational research (Brews & Hunt, 1999; Homburg et al., 1999; Pearce, Robbins, & Robinson, 1987; Priem, Rasheed, & Kotulic, 1995) and, for instance, Dess and Robinson (1984) find a strong correlation between subjective and objective performance measures.

#### 3.2.1 Trust

To measure the level of trust that a franchisor holds towards the franchisees, we employed a four‐item 7‐point Likert scale based on Anderson and Narus (1990) and Dyer and Chu (2000). Franchisors were asked to assess the following items on a 7‐point Likert scale ranging from 1 (*strongly disagree*) to 7 (*strongly agree*): “There is an atmosphere of openness and honesty between us and the partners,” “The exchange of information between us and the partners goes beyond the agreed scope,” “There is great trust between ourselves and the partners,” and “The cooperation is based on partnership basis.”

#### 3.2.2 Environmental uncertainty

Environmental uncertainty represents difficulty in anticipating changes in the environment surrounding the company and its activity (Sheng et al., 2006). The level of environmental uncertainty was measured with a three‐item Likert scale based on Celly and Frazier (1996) and John and Weitz (1988). Respondents were asked to assess the following items on a 7‐point Likert scale ranging from 1 (*strongly disagree*) to 7 (*strongly agree*): “It is very difficult to predict the market development,” “The sales at the outlet level are very fluctuating,” and “The market environment in the local market is changing rapidly.”

#### 3.2.3 Intangible system‐specific assets

Intangible system‐specific assets refer to the franchisor's knowhow that is embedded in the company's organizational routines and employees. The system knowledge is to varying extent tacit and difficult to transfer to network partners so that the franchisees can replicate it at the local markets (Madhok, 1997; Nonaka, 1994). Based on Erramilli et al. (2002), we employ a 7‐point Likert‐type scale to measure intangible system‐specific assets based on the nontransferability of system‐specific knowhow in various areas (1 = *low nontransferability*; 7 = *high non‐transferability*). Franchisors were asked to assess the nontransferability of the system‐specific knowhow of the following knowhow areas (see Table 2): marketing knowhow, organizational knowhow, administrative knowhow, quality management knowhow, accounting knowhow, and IT‐knowhow.

**Table 2:** Measurement model^a^^,^ b ^,^ c

	Standardized regression weight	Composite reliability	Average variance extracted
Performance			
Reduction of operational costs	0.72 (0.31)	0.74	0.49
Increase in innovation	0.50		
Savings in control and coordination costs	0.84 (0.31)		
Franchisor trust			
Cooperation on partnership basis	0.75	0.89	0.67
Exchange of information beyond agreed scope	0.63 (0.16)		
Trust between ourselves and partners	0.92 (0.13)		
Atmosphere of openness and honesty between partners	0.94 (0.12)		
Environmental uncertainty			
Market environment is changing rapidly	0.45 (0.10)	0.76	0.53
Difficult to predict market development	0.87 (0.14)		
Sales at the outlet level is very fluctuating	0.80		
Franchisees‘transaction‐specific investments			
Franchisees‘expenses for initial training	0.78	0.78	0.64
Franchisees' expenses for initial technical and organizational support	0.82 (0.22)		
Intangible system‐specific assets (operationalized by the degree of nontransferability)			
IT knowhow	0.66 (0.09)	0.92	0.65
Accounting knowhow	0.76 (0.08)		
Quality management knowhow	0.78 (0.08)		
Administrative knowhow	0.90 (0.06)		
Organizational knowhow	0.93		
Marketing knowhow	0.76 (0.08)		
Intangible brand name assets			
Brand is very important for achieving competitive advantage in terms of recognition and strength as compared to the competitors	0.64 (0.10)	0.85	0.60
Franchise system enjoys a good reputation for quality	0.87 (0.09)		
Franchise system enjoys high brand recognition as compared to competitor	0.88		
Brand name is strong as compared to competitor	0.68 (0.10)		

a 123 observations.

b Standard errors in parentheses.

c All items are statistically significant (*p* < .001) determinants of the latent variables.

#### 3.2.4 Intangible brand name assets

To assess the perceived brand reputation of the franchise system, we adapted the scale from Barthélemy (2008). Franchisors were asked to assess the following items on a 7‐point Likert scale ranging from 1 (*strongly disagree*) to 7 (*strongly agree*): “Our brand name is very strong as compared to our competitors,” “Our franchise system enjoys higher brand recognition as compared to our competitors,” “Our franchise system enjoys a good reputation for quality,” and “Our brand name is very important for achieving competitive advantage in terms of recognition and strength compared to their competitors.”

#### 3.2.5 Franchisees' transaction‐specific investments

According to the transaction cost theory, the choice of governance form is influenced by transaction‐specific investments (Klein, 1996). We applied a two‐item scale from Stump and Heide (1996) and Heide and John (1990) to assess the extent of specific investments that franchisees make when they enter the franchise relationship. The original scale items were adapted to the franchise context. Franchisors were asked to rate the following questions on a 7‐point Likert scale (1 = *not at all*; 7 = *to a very high extent*): “To what extent does the franchisee bear the initial training costs?”; “To what extent does the franchisee bear the initial technical and organizational support?”

#### 3.2.6 Franchisor performance

To assess performance, franchisors were asked the question “To what extent have you realized the following goals in the last year?”, to rate the performance with a three‐item 7‐point Likert scale: “Reduction in operational costs, savings in coordination and control costs, and increase in innovation.” Reduction in operational costs and savings in coordination and control costs refer to the exploitation efficiency and increase in innovation to the exploration efficiency of the network (Ghemawat & Ricart Costa, 1993).

### 3.3 Results

#### 3.3.1 Measurement model

The measurement model refers to constructing latent construct variables from the observed indicator variables. To specify the measurement model, an iterative process based on content and statistical considerations was used (Anderson & Gerbing, 1988). During this process, six items were eliminated from the model because they failed to exhibit appropriate validity or reduced the quality of the measurement model (“franchisees' expenses for the equipment” from the transaction‐specific investments measure; “System growth” and “increase of sales revenues” from the performance measure; “The outlet sales predictions are usually very precise” from the environmental uncertainty measure; and “brand name” and “controlling knowhow” from the system‐specific measure). We assessed the adequacy of the measurement model by examining individual item reliability, convergent validity, and discriminant validity.

#### 3.3.2 Reliability and convergent validity

First, we assessed the individual items. All of the item loadings are significant (*p* < .001) and exhibit factor loadings ≥0.4 (Ford, MacCallum, & Tait, 1986; Hitt, Hoskisson, Johnson, & Moesel, 1996), providing support for the measurement model. Second, we calculated the composite reliability, a measure of internal consistency similar to Cronbach's alpha drawing on standardized loadings and measurement error (Fornell & Larcker, 1981; Nunnally, 1978). For each latent variable, the composite reliability values exceed the suggested threshold of 0.7 indicating satisfactory internal consistency (Fornell & Larcker, 1981; Nunnally, 1978). Third, we calculated the average variance extracted that compares the amount of variance captured by the construct with the amount of variance due to measurement error (Fornell & Larcker, 1981). Except for one latent variable, which is slightly below the suggested threshold of 0.5, all constructs exhibit a satisfactory ratio. Table 2 presents the results of the discussed analyses. Finally, we assessed overall convergent validity with the comparative fit index. The comparative fit index is a relative fit index estimating the percentage of variation explained by the proposed model compared with the independence model. The suggested threshold value of 0.9 signifies that the measurement model explains 90% more variance than the model of complete independence and is exceeded in our study (Bentler, 1989; see Table 3). Similarly, the root mean squared error of approximation and the standardized root mean square residual also indicate that the measurement model exhibits an acceptable fit (Hu & Bentler, 1999; see Table 3).

**Table 3:** Goodness‐of‐fit measurement model

	M1 Single factor model	M2 Single intangible construct	M3 Original model
${Χ}_{\mathrm{df}}^{2}$	1106.59_194_ (*p* < .01)	536.93_199_ (*p* < .01)	299.91_194_ (*p* < .01)
CFI	0.39	0.77	0.93
RMSEA	0.19	0.12	0.07
SRMR	0.07	0.13	0.19
AIC	1194.58	644.93	417.91
BCC	1215.03	670.02	445.32
BIC	1318.32	796.79	583.83

Abbreviations: AIC, Akaike information criterion; BCC, Browne–Cudeck criterion; BIC, Bayes information criterian; CFI, comparative fit index; RMSEA, root mean squared error of approximation; SRMR, standardized root mean square residual.

#### 3.3.3 Discriminant validity

Discriminant validity tests to which extent the different constructs are distinct. We first assessed discriminant validity by verifying that the variance shared between constructs is less than the variance shared between a constructs and its measures (Fornell & Larcker, 1981). As can be seen in Table 4, the diagonal elements, the square root of the average variance extracted of the focal constructs, are greater than the off‐diagonal elements in the corresponding rows and columns, the correlation between the focal constructs, and all other constructs, indicating adequate discriminant validity. Second, we investigated whether collapsing theoretically related factors result in a substantial loss of model fit (Anderson, 1987; Bagozzi & Phillips, 1982). If the loss of model fit is not substantial, the theoretically proposed factor does not exhibit sufficient discriminant validity. Following standard procedure, we compared three models of different aggregation levels. Table 3 shows the comparison between a single factor model (M1) in which all items are assumed from one theoretical construct, a model in which the two most closely related latent variables—the two variables addressing intangible assets—have been collapsed (M2), and the original measurement model with the seven distinct latent variables as proposed in theory (M3; see the description of the different variables employed in the study in the Measurement section). Initial evidence for discriminant validity is provided when the comparative fit index of a model with more constructs falls by more than 1% (Widaman, 1985), which is given in all instances. Following Arbuckle (2011), we compared the Bayes information criterian, the Browne–Cudeck criterion, and the Akaike information criterion. Again, the proposed model outperforms the alternative models providing support for discriminant validity.

**Table 4:** Descriptive statistics and correlation of constructs

	(1)	(2)	(3)	(4)	(5)	(6)
(1) Performance	0.70					
(2) Knowledge‐based trust	0.35	0.82				
(3) Environmental uncertainty	−0.42	−0.36	0.73			
(4) Transaction‐specific investments	0.39	−0.05	0.04	0.80		
(5) Intangible system specific assets	0.42	0.02	0.01	0.40	0.80	
(6) Intangible brand name assets	0.44	0.30	−0.17	0.22	0.16	0.77
Mean	4.38	5.78	3.72	4.05	3.64	5.60
Standard deviation	0.98	1.05	1.37	1.48	1.32	1.13
Min	1.6	1	1	1	1	1.3
Max	7	7	7	7	6.6	7

We also performed pairwise comparisons between other latent constructs leading to comparable results. Overall, the measurement model can be considered acceptable despite the significant chi‐square statistic, given the other supportive indexes (Anderson & Gerbing, 1988). Assessment of the individual items and latent variables provided support for adequate reliability and convergent validity as well as discriminant validity.

#### 3.3.4 Mediation analysis

In intervening variable analyses, the causal variables *X*_n_ (intangible brand name assets, intangible system‐specific assets, franchisees' transaction‐specific investments, and environmental uncertainty) are hypothesized to affect the outcome variable *Y* (performance) via a mediating variable *M* (trust; see Figure 1). The direct effects of *X*_n_ on performance (path *c*') are tested in the hypotheses H2a to H5a, and the effects of *X*_n_ on the mediating variable trust (path *a*) are tested in the hypotheses H2b to H5b. The effect of trust on performance (path *b*) is tested in H1. Finally, the indirect effects of *X*_n_ on performance are tested in the mediation hypotheses H2b to H5b. The total effect *X*_n_ have on performance is constituted by the direct and the indirect effect (*c* = *ab* + *c*'). 1The total effect *c* is identical with *β* of *X*_n_ a regression model without controlling for trust and *c*' when controlling for trust in the model.


To conduct our tests for mediation and indirect effects, we utilize PROCESS (Hayes, 2012) and use bootstrapping to calculate confidence intervals and test the indirect effects (*ab*). As opposed to the Sobel test, bootstrapping does not require the assumption of normality of the sampling distribution, which is often violated by the indirect effect *ab* (Hayes, 2009). Furthermore, simulations show that bootstrapping is among the more valid and powerful approaches for testing the effect of an intervening variable (Hayes, 2009; MacKinnon, Lockwood, & Williams, 2004; Williams & MacKinnon, 2008). As recommended by Edwards and Lambert (2007), we generated 95 and 99% bias corrected bootstrapped confidence intervals using 10,000 bootstrap samples. The results of the intervening variable model are shown in Table 5. Table 5 reports the mediation test statistics including the indirect effects, total effects, and the indirect effect ratios.

**Table 5:** Mediation test statistics^a^^,^ b

Independent variables (*X*_*n*_)	Direct effect on performance (*c'*)	Effect on trust (*a*)	Effect of trust on performance (*b*)	Indirect effect (*ab*)	Total effect (*c* = *c*' + *ab*)	Relative Indirect Effect (P_M_)^c^	Relative Indirect Effect (P_D_)^d^
Environmental uncertainty	−0.13*** (0.05)	−0.20*** (0.07)	0.20*** (0.07)	−0.04*** (0.02)	−0.17*** (0.05)	0.24** (4.01)	0.32** (221.84)
Transaction‐specific investments	0.15*** (0.05)	−0.07 (0.07)		−0.01 (0.02)	0.14** (0.05)	−0.10 (3.47)	−0.09 (2.05)
Intangible system‐ specific assets	0.21*** (0.06)	0.05 (0.07)		0.01 (0.02)	0.22*** (0.06)	0.04 (0.13)	0.04 (0.16)
Intangible brand name assets	0.19*** (0.07)	0.25*** (0.08)		0.05** (0.03)	0.24*** (0.07)	0.21** (0.62)	0.26** (5.18)

a Standard errors in parentheses.

b *** p < .01; ** *p* < .05; * *p* < .1

c $P_{M}=\frac{\mathrm{ab}}{\mathrm{ab}+c^{\prime }}=\frac{\mathrm{ab}}{c}=1-\frac{c^{\prime }}{c}$

d $P_{D}=\frac{\mathrm{ab}}{c^{\prime }}=\frac{\mathrm{ab}}{c-\mathrm{ab}}=\frac{c}{c^{\prime }}-1$

#### 3.3.5 Direct effects on performance

**Figure 3:**
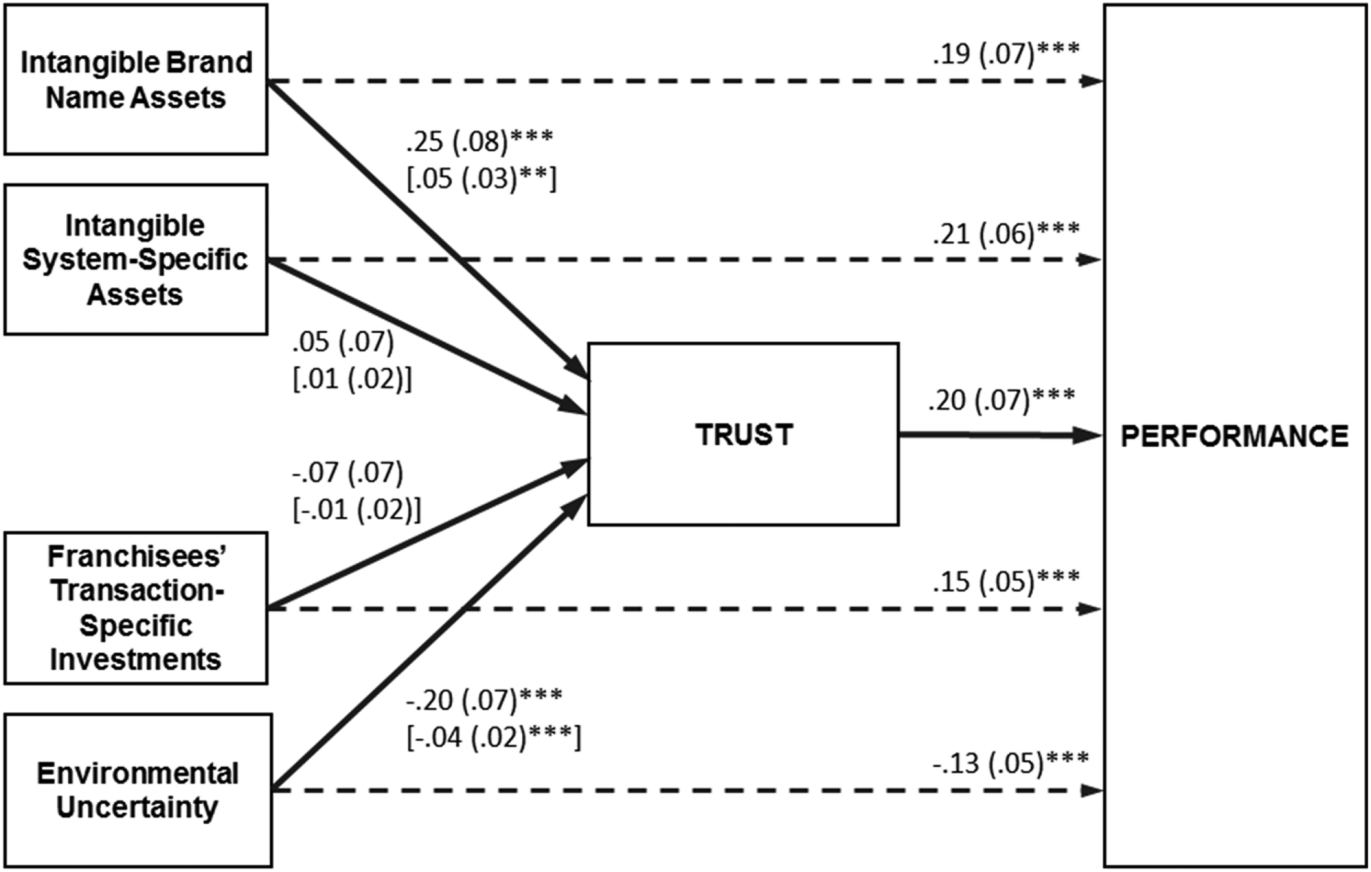
Results of the Hypotheses Test

In a first step, it is important to assess whether the mediator *M* is significantly related to the outcome variable *Y* (path *b*, H1) and *X*_n_ have a significant effect on trust (path *a*, H2a to H5a). Our results confirm H1 and show a positive and significant effect of franchisor trust on the performance of the franchise system (*b* = 0.20, *p* < .01; see Figure 3 & Table 5). Thus, the higher the level of franchisor's trust toward the franchisee, the better the performance of the franchise system (Dyer & Chu, 2003; Katsikeas et al., 2009; Robson, Katsikeas, & Bello, 2008). We also find significant direct effects for transaction cost as well as knowledge‐based variables confirming the hypotheses H2a, H3a, H4a, and H5a. Whereas environmental uncertainty has a negative effect on the performance of franchise firms (*c*' = −0.13, *p* < .01), franchisees' transaction‐specific investments (*c*' = 0.15, *p* < .01), intangible system specific assets (*c*' = 0.21, *p* < 0.1), and intangible brand name assets (*c*' = 0.19, *p* < .1) affect performance positively (see Figure 3 & Table 5). Overall, our results show that all predicted direct effects on performance are significant confirming the hypothesized relationships.

#### 3.3.6 Direct effects on trust and indirect effects on performance

More importantly in the context of the present study, we find that environmental uncertainty and intangible brand name assets additionally affect the performance of franchise firms via trust (Figure 3 & Table 5). Results show that the higher the environmental uncertainty, the lower the level of trust in the franchisees (*a* = −0.20, *p* < .01; see Figure 3 & Table 5; H2b). Consistently, we also find a significant negative indirect effect (*ab* = −0.17, *p* < 0.01; see Table 5; H2c). Thus, environmental uncertainty has not only a direct adverse effect on the performance of the franchise system but additionally affects performance in a detrimental manner by reducing the level of franchisors' trust towards franchisees. Conversely, intangible brand name assets exhibit a positive direct effect on trust (*a* = 0.25, *p* < 0.01; see Figure 3 & Table 5; H5b) as well as a significant and positive indirect effect on performance via trust (*ab* = 0.05, *p* < .05; see Figure 3 & Table 5; H5c). Intangible brand name assets foster franchisor's trust in the franchisee, which in turn increases performance. Thus, for both environmental uncertainty (*ab* + *c*' = −0.17, *p* < .01; see Table 5) and intangible brand name assets (*ab* + *c*' = 0.24, *p* < .01; see Table 5), the total effect on performance is composed of the direct effect on performance (*c*') as well as the indirect effect (*ab*) via trust. Contrary to our hypotheses, franchisees' transaction‐specific investments (H3b & H3c) and intangible system‐specific assets (H4b & H4c) exhibit neither a direct effect on trust nor an indirect effect on performance via trust (see Figure 3 & Table 5). Although transaction‐specific investments and intangible system‐specific assets have a direct effect on performance as hypothesized, the relationship between the two variables and performance is not mediated by trust.

We additionally calculated *P*_*M*_ (the ratio of the indirect to the total effect) and *P*_*D*_ (the ratio of indirect to direct effect; Alwin & Hauser, 1975; MacKinnon, 1994). The two measures reflect the relative magnitude of the mediation effect. *P*_*M*_ represents the percentage of the total effect that is mediated, hence stating how much of the total effect can be accounted for by the mediation path. *P*_*D*_ measure indicates the relative size of the mediating effect to the direct effect. Although these measures have to be interpreted with caution (MacKinnon, Warsi, & Dwyer, 1995), they provide an indication of the strength of the mediating effect. In our study, we find that the indirect effect is weaker than the direct effect for both environmental uncertainty (*P*_*D*_ = 32%) as well as for intangible brand name assets (*P*_*D*_ = 26%) (see Table 5). Nevertheless, the mediating effect of environmental uncertainty (*P*_*M*_ = 24%) and intangible brand name assets (*P*_*M*_ = 21%) explains almost a quarter of the total effect these two variables have on the performance of the franchise network.

Overall, our results confirm that trust is a central mediator on the performance relationship for environmental uncertainty and intangible brand name assets—they affect the performance of the franchise system not only directly but also by increasing or decreasing franchisor's trust in the franchisees.

## 4 DISCUSSION AND IMPLICATIONS

In this study, we examine the dual role of trust by developing a research model that explains the mediating effect of trust on franchisor performance. Specifically, we argue that trust has both a “bright side” and a “dark side” in franchisor–franchisee relationships by showing that intangible knowledge assets have a positive indirect effect on performance via trust, due to its positive knowledge exchange effect, and environmental uncertainty has a negative indirect effect on performance via trust, due to its relational risk‐increasing effect. The data from the franchise sector in Germany indicate that trust positively mediates the impact of intangible brand name assets and negatively mediates environmental uncertainty on franchisor performance.

First, consistent with the transaction cost theory (Dyer & Chu, 2000, 2003) and the knowledge‐based view (Nonaka, 1994; Zander & Kogut, 1995), the results support the positive direct effect of trust, franchisees' transaction‐specific investments, intangible brand name and system‐specific assets, as well as the negative direct effect of environmental uncertainty on franchisor performance. Specifically, our results confirm the hypothesis that higher levels of knowledge‐based trust of the franchisor towards the franchisees increase the performance of the franchise. This positive effect can be attributed to reducing transaction costs due to relational risk reduction and increasing the relational rents due to a higher level of knowledge sharing between the franchisor and the franchisees (Dyer & Singh, 1998; Gorovaia & Windsperger, 2011; Zajac & Olsen, 1993). In addition, our results show that franchisees' transaction‐specific investments have a strong positive impact on performance, supporting the view that transaction‐specific investments at the local market increase the relationship‐specific quasi‐rents and hence the residual income stream of the franchisor and franchisees. Furthermore, the results indicate that intangible brand name assets and system‐specific knowhow as source of competitive advantage have a positive effect on performance. On the other hand, high environmental uncertainty has a negative effect on franchisor performance. This is due to the fact that high environmental uncertainty at the local markets increases franchisor's information processing requirements and hence the information and communication costs between the headquarters and the local outlets.

Second, the data provide support of the mediator hypotheses of trust regarding the indirect effect of environmental uncertainty and intangible brand name assets on franchisor performance. These results support the “dark side” and “bright side” of trust effects. Environmental uncertainty has a negative indirect performance effect by reducing the level of franchisor trust towards their franchisees. This may arise in a situation where high local market uncertainty increases the franchisor's relational risk by being unable to assess franchisees behavior and performance. In addition, because franchisors' vulnerability due to strategic inflexibility increases when the franchise partners operate in an uncertain local market environment, their level of trust is expected to be lower. Consistent with the knowledge‐based hypothesis, we find support of the positive indirect effect of intangible brand name assets on performance. This finding is in line with Hoetker and Mellewigt (2009), who argue that relational mechanisms play a critical role in transferring and exploiting knowledge‐based assets. A strong brand name is associated with higher levels of trust, as franchisor's investments in intangible brand assets signal commitment and thus enhance trust in the franchisee relationship. On the other hand, the results of the study do not support the indirect effect of franchisees' transactions‐specific investments and intangible system‐specific assets on franchisor performance. One reason why the indirect effect of franchisees' transaction‐specific investments is not significant might be due to the fact that the bonding effect is more important for the direct performance effect of transaction‐specific investments and is less important for the relational risk effect via trust on performance. In addition, the nonsignificant result of the indirect performance effect of intangible system‐specific assets might result from the specific operationalization of the construct, which is based on Erramilli et al. (2002).

The article has implications both for research and practice. This study extends the relational governance literature in franchising by investigating the dual role of franchisor trust tested as mediator between transaction cost and knowledge‐based variables and performance in franchising networks. Trust negatively mediates the impact of environmental uncertainty on performance. Therefore, the “dark side” of trust due to strategic blindness is especially important under high environmental uncertainty resulting in a negative performance effect. Conversely, trust positively mediates the impact of intangible brand name assets on performance. Hence, the “bright side” of trust as facilitator of knowledge exchange is particularly strong under intangible knowledge assets resulting in an increase of franchisor's relationship‐specific rents and performance. Overall, we can conclude that our results highlight the central role of trust as it acts as mediator for other franchise relevant variables. As shown in this study, intangible brand name assets and environmental uncertainty do not only have a direct impact on the performance of the franchise system but also affect the level of trust. As trust also influences performance, it exacerbates the positive effects of intangible brand name assets and the negative effects of environmental uncertainty on performance. This implies that the negative effect of uncertainty on trust and performance can be mitigated by employing levers that affect trust and performance such as intangible brand name assets.

This study bears important managerial implications for the management of the franchising network. The franchisor has to consider the importance of trust as performance‐enhancing relational factor that increases with the intangibility of knowledge assets. This indicates that the management of a franchise system with highly intangible brand name assets should particularly focus on trust and close cooperation between franchisor and franchisees. At the same time, the franchisor should be cautious to retain entrepreneurial responsiveness by reducing the „hidden costs“of trust (Selnes & Sallis, 2003, 84), when the local market environment is highly uncertain (Krishnan et al., 2006). For instance, if the franchising network is characterized by a high degree of local market uncertainty and a low degree of intangibility of brand name assets, the adverse performance effect of trust, due to high environmental uncertainty, will likely exceed the positive performance effect of brand name assets via trust. Under such circumstances, our results suggest that franchisors are advised to invest in and implement trust enhancing mechanisms. By doing so, the negative effects of environmental uncertainty on performance via trust can be mitigated. Consequently, contrary to the common practice in franchise relationship management, franchisor's decision making has to take into account the hidden costs of high trust. Higher levels of franchisor trust in the network partners may results in problems of strategic inflexibility, in particular, when entrepreneurial responsiveness and knowledge exploration are very important under high environmental uncertainty.

A major limitation of the study is the usage of cross‐sectional data. Whereas the presented theory proposes causal linkages, the empirical results are strictly speaking only correlational. For instance, the positive effect of trust on performance investigated in the present study has been theoretically elaborated and empirically shown in previous research. However, a bidirectional effect is also plausible. In order to strengthen our causal argument and isolate the causal effect of trust on performance, future research would be required. In addition, because the mediating role of trust between system‐specific assets and performance was not confirmed by our data, future studies should test this relationship with alternative measures of intangible system‐specific assets.

We believe further research should address the dual role of trust in franchise relationships, in particular, by examining the trade‐off between the “dark side” effect of high trust relationships will likely dominate the “dark side” effect of high trust relationships, because high trust franchisor–franchisee relationships facilitate efficient knowledge sharing under highly intangible knowhow. Conversely, if a franchise network is characterized by high environmental uncertainty and less intangible brand name assets, the “dark side” effect will likely dominate the “bright side” effect of high trust relationships, because high trust franchisor–franchisee relationships will result in strategic blindness by inhibiting exploration of novel information in the external environment. Therefore, if the environmental uncertainty is high and the franchisor's brand name assets are less intangible, the transfer of less intangible system knowhow to the local franchisees does not require a high level of trust. Simultaneously, high environmental uncertainty requires high entrepreneurial flexibility of the franchisor and hence a lower level of trust in his network partners to flexibly respond to novel information. Consequently, future research should conduct case study analysis by comparing franchise systems with low levels of environmental uncertainty and high levels of intangible knowledge assets with franchise systems with high levels of environmental uncertainty and low levels of intangible assets.
